# Inhibition of IKKβ/NF-κB signaling pathway to improve Dasatinib efficacy in suppression of cisplatin-resistant head and neck squamous cell carcinoma

**DOI:** 10.1038/s41420-020-0270-7

**Published:** 2020-05-15

**Authors:** Zejia Yang, Jipei Liao, Kevin J. Cullen, Hancai Dan

**Affiliations:** 1 Marlene and Stewart Greenebaum Comprehensive Cancer Center, University of Maryland School of Medicine, Baltimore, MD USA; 2 Department of Pathology, University of Maryland School of Medicine, Baltimore, MD USA

**Keywords:** Targeted therapies, Translational research

## Abstract

Proto-oncogene tyrosine-protein kinase Src plays an important role in Head and Neck Squamous Cell Carcinoma (HNSCC). However, the FDA-approved SRC inhibitor Dasatinib shows very limited efficacy in HNSCC clinical trials, even though Dasatinib can completely inhibit SRC in the laboratory setting. These results suggest that SRC inhibition can cause compensatory up-regulation and/or activation of other survival pathways, which suggests that co-targeting of SRC and the potential signaling pathways may improve the Dasatinib efficacy. In this study, we investigated the role of IKKβ/NF-κB in regulation of the sensitivity of cisplatin-resistant HNSCC to Dasatinib. Additionally, we wished to determine whether inhibition of the IKKβ/NF-κB signaling pathway could enhance Dasatinib efficacy to inhibit cisplatin-resistant HNSCC without the use of cisplatin. Previous studies have shown that ETS-1 is a crucial SRC effector protein that regulates cancer cell proliferation, anti-apoptosis, and metastasis. We found that SRC kinase inhibition by Dasatinib decreased ETS-1 expression but caused elevation of IKKβ/NF-κB signaling in multiple cisplatin-resistant HNSCC. Interestingly, inhibition of IKKβ/NF-κB by CmpdA (Bay65-1942), a recently identified IKKβ inhibitor, also led to a decrease in ETS-1 levels. Moreover, the knockdown of IKK, but not NF-κB, dramatically decreased ETS-1 expression. In addition, IKKβ and ETS-1 interacted in cisplatin-resistant HNSCC. These data demonstrated cross-talk between SRC and IKK to regulate NF-κB and ETS-1. Furthermore, we found that simultaneous inhibition of SRC and IKKβ through a Dasatinib and CmpdA combination synergistically inhibited NF-κB activation and ETS-1expression, suppressed cell proliferation, and induced apoptosis. Taken together, our data indicate that SRC and IKKβ play crucial roles in cisplatin-resistant HNSCCC and co-targeting SRC and IKKβ could be an effective strategy to treat cisplatin-resistant HNSCC.

## Introduction

Head-and-neck cancer originates in organs such as the larynx, pharynx, lips, mouth, nose, and salivary glands. Since most head-and-neck cancers begin in the squamous cells of these organs, these cancers are named head-and-neck squamous cell carcinoma (HNSCC). HNSCC is currently the sixth cause of cancer-related deaths in the world and accounts for about 3 percent of all cancers in the United States^1–3^. The main treatments for HNSCC include surgery, radiation therapy, chemotherapy, and immunotherapy, which are used singly or in combination for different stages of disease. Surgery or radiation therapies have proven very successful for treatment of early-stage HNSCC patients and, in combination with radiation therapy, achieve good survival rates in patients who develop loco-reginal lymphoma metastasis. For all metastatic HNSCC and most recurrent HNSCC, however, chemotherapy is the only treatment option^4–8^.

Cisplatin has been a major anti-cancer drug used in head-and-neck cancer therapy and is frequently combined with other chemotherapy drugs, such as Taxol and 5-Fluorouracil (5-FU)^3,9,10^. Patients initially show a good response to cisplatin-combined chemotherapy, but nearly all patients eventually develop resistance to cisplatin and die within a year. In order to explore new therapies for cisplatin-resistant HNSCC, it is vital to define the mechanisms that confer cisplatin resistance and identify effective inhibitors to block crucial survival-signaling pathways that are elevated or activated upon cisplatin treatment^11,12^.

Proto-oncogene tyrosine-protein kinase SRC plays an important role in HNSCC. It has been reported that, when activated, SRC promotes tumorigenesis through its downstream signaling pathways, including PI3 kinase/Akt/mTOR and MEK/ERK, to facilitate cancer growth, migration, invasion, and metastasis, as well as chemotherapy resistance^13–15^. Therefore, SRC kinase has been an attractive target for cancer therapy, including those for HNSCC. However, the FDA approved SRC inhibitor Dasatinib shows very limited efficacy in HNSCC clinical trials, even though it completely inhibits SRC in the laboratory setting^16–18^. This suggests that SRC inhibition can cause compensatory up-regulation and/or activation of other survival pathways, which means co-targeting SRC and other signaling pathways could improve Dasatinib efficacy.

Our long-term goal is to discover new therapeutics to treat cisplatin-resistant HNSCC through targeted therapies without the use of cisplatin. Patients who have progressed to cisplatin-resistant HNSCC may not continue to tolerate increasing doses of cisplatin. We previously reported that elevated IKKβ/NF-κB activity played an important role to control cell proliferation and cisplatin resistance^19^. Recently, we found that SRC signaling pathways were also up-regulated in cisplatin-resistant HNSCC, and SRC kinase regulated cisplatin-resistant HNSCC through regulation of transcription factor ETS-1^20^. These results prompted us to investigate the molecular link between SRC and IKKβ/NF-κB in cisplatin-resistant HNSCC regulation.

In the current study, we found that treatment of cisplatin-resistant HNSCC with SRC inhibitor, Dasatinib, inhibited the SRC/ETS-1 signaling pathway, thereby leading to further elevation of the IKKβ/NF-κB pathway. Moreover, we found that IKKβ interacted with ETS-1 to regulate its degradation in a manner independent of SRC and NF-κB. Therefore, SRC and IKKβ regulated ETS-1 in parallel. Subsequently, a combination of the SRC inhibitor, Dasatinib, and the IKKβ inhibitor, CmpdA, led to complete inhibition of SRC, IKKβ, NF-κB, and ETS-1. Consistently, this combination synergistically suppressed cell proliferation and induced apoptosis.

## Results

### Inhibition of SRC and ETS-1 and induction of IKKβ/NF-κB by the SRC inhibitor Dasatinib in cisplatin-resistant HNSCC

**Fig. 1:**
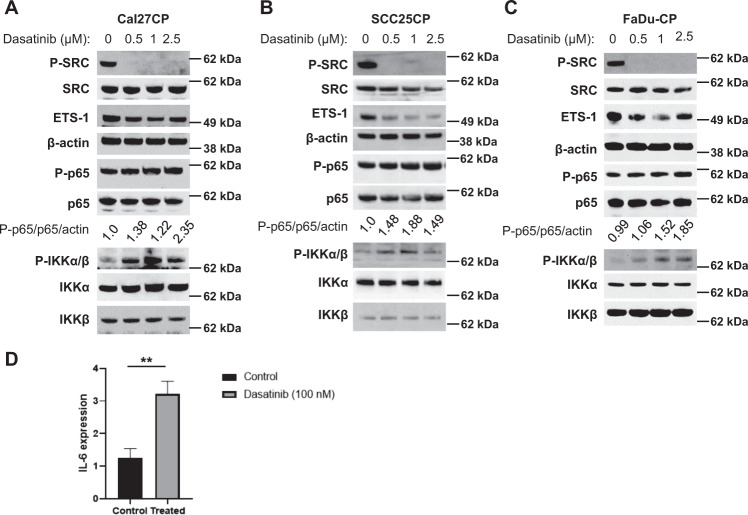
Dasatinib inhibits SRC and ETS-1 but induces IKKβ/NF-κB in cisplatin resistant HNSCC. **a**–**c** Cell lysates were prepared from Cal27CP (**a**), SCC25C (**b**), and FaDu-CP (**c**) cells treated with Dasatinib for 8 h and phosphorylation and total levels of SRC, p65, IKK, and expression of ETS-1 and β-actin were detected by Western blot analysis. The density of P-p65, p65, and β-actin bands was quantitated and the level of phosphorylation of p65 was normalized by p65 and β-actin. **d** Cal27CP cells were treated with DMSO or 200 nM Dasatinib for 24 h, mRNA was extracted and the expression of IL-6 was detected by real time PCR.

We recently showed that Dasatinib treatment inhibited phosphorylation of SRC and decreased ETS-1 expression in cisplatin resistant HNSCC cells^20^. Here, we wanted to determine the effect of Dasatinib treatment on IKKβ/NF-κB pathway activity. Consistent with our previous data, Dasatinib completely inhibited phosphorylation of SRC and led to a modest decrease of ETS-1 expression in cisplatin-resistant Cal27CP cells (Fig. 1a). Interestingly, phosphorylation of NF-κB at Serine 536 and phosphorylation of IKKα/β at their activation loop increased, whereas the total levels of these proteins did not change with Dasatinib treatment (Fig. 1a). Similar results were found in cisplatin-resistant SCC25CP cell line originated from cisplatin-sensitive SCC25 cells and cisplatin-resistant FaDu-CP cell line originated from cisplatin-sensitive FaDu cells (Fig. 1b, c). In order to confirm that IKK/NF-κB activity is elevated by Dasatinib treatment, we tested the gene expression of IL-6, a NF-κB target gene^21,22^. The results showed that Dasatinib significantly elevated IL-6 expression in Cal27CP cells (Fig. 1d). Our data indicate that Dasatinib inhibits SRC/ETS-1 expression but induces the IKK/NF-κB pathway in cisplatin-resistant HNSCC.

### IKKβ inhibitor, CmpdA, treatment also inhibits ETS-1 expression

**Fig. 2:**
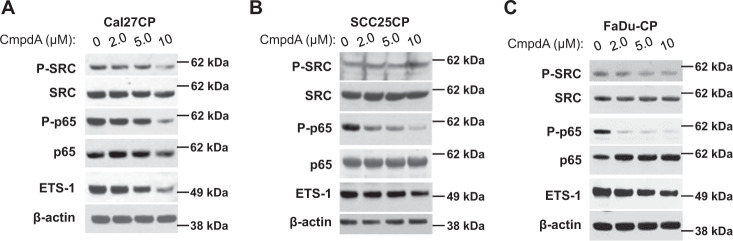
Inhibition of IKKβ decreased ETS-1 expression. Cal27CP (**a**), SCC25C (**b**), and FaDu-CP (**c**) cells were treated with increasing concentrations of IKKβ inhibitor, CmpdA, for 24 h and phosphorylation and total levels of SRC, p65, and expression of ETS-1 and β-actin were detected by Western blot analysis.

We treated Cal27CP, SCC25CP, and FaDu-CP cells with the IKKβ inhibitor, CmpdA (Bay65-1942)^23^, for 24 h and examined its effect on ETS-1 expression. CmpdA decreased phosphorylation of p65 in a dose-dependent manner, which indicated that IKK activity was inhibited. As predicted, CmpdA also decreased ETS-1 expression in a dose-dependent manner but had no effects on SRC phosphorylation (Fig. 2a–c). Our results confirm that ETS-1 is regulated by IKK through a mechanism dependent or independent of NF-κB in cisplatin resistant HNSCC cells.

### Depletion of IKK, but not NF-κB, decreases ETS-1 expression in cisplatin-resistant HNSCC

**Fig. 3:**
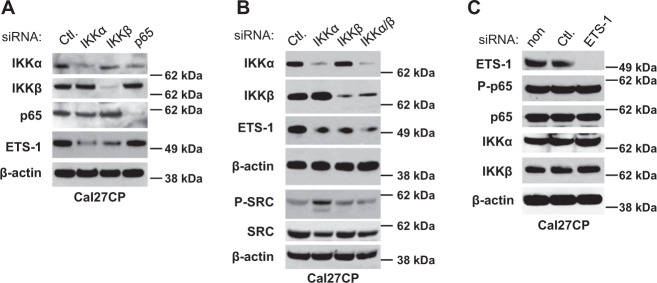
IKK regulates ETS-1 independent of NF-κB. **a** Knockdown of IKKα or IKKβ, but not NF-κB, decreased ETS-1 expression. Cal27CP cells were transfected with non-target siRNA, IKKα, IKKβ, or p65 for 72 h and expression of IKKα, IKKβ, p65, ETS-1, and β-Actin were detected by Western blot analysis. **b** Both IKKα and IKKβ are involved in regulation of ETS-1 expression. Cal27CP cells were transfected with non-target siRNA, IKKα, IKKβ, or IKKα plus IKKβ for 72 h and expression of IKKα, IKKβ, ETS-1, and β-Actin were detected by Western blot analysis. **c** Knockdown of ETS-1 had no effects on p65 phosphorylation. Cal27CP cells were transfected with non-target siRNA or siRNA ETS-1 for 72 h and expression of IKKα, IKKβ, ETS-1, phospho-p65, p65, and β-actin were detected by Western blot.

In order to determine the molecular link between the IKKβ/NF-κB pathway and ETS-1, we used siRNA against IKKα, IKKβ, or NF-κB (p65) to decrease their expression, respectively, before examining their effects on ETS-1 expression in Cal27CP cells. Western blot results showed that these siRNA were effective at lowering the expression of their target proteins. As shown in Fig. 3a, knockdown of IKKα or IKKβ, but not p65 (NF-κB), led to decreased ETS-1 expression (Fig. 3a). Furthermore, we found that concurrent knockdown of IKKα and IKKβ caused more significant decreases of ETS-1 compared to knockdown of either IKKα or IKKβ alone. These results suggested that both IKKα and IKKβ are involved in the regulation of ETS-1 expression (Fig. 3b) and IKKα and IKKβ regulation of ETS-1 is NF-κB independent. Moreover, we found that ETS-1 knockdown had no effect on NF-κB phosphorylation and IKK expression (Fig. 3c). Therefore, ETS-1 can act as a downstream target of IKK.

### IKKβ interacts with ETS-1 and regulates its degradation

**Fig. 4:**
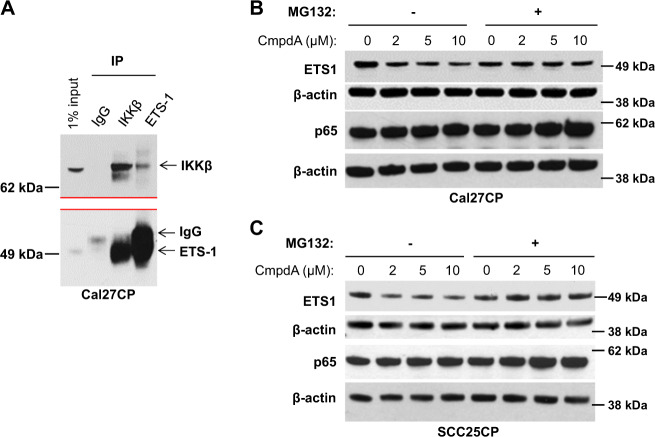
IKKβ associates with ETS-1 and regulates its degradation. **a** The lysates from Cal27CP cells were immunoprecipitated with anti-IKKβ, anti-ETS-1, or IgG control, electrophoresed on an SDS gel, and detected with IKKβ and ETS-1 antibodies, respectively. *Note*: the red lines in panel *A* showed that blots were cut for detection of IKKβ (upper) and ETS-1 (bottom), respectively. **b**, **c** Cal27CP (**b**) and SCC25CP (**c**) cells treated with DMSO control or MG-132 for 2 h were incubated with media containing increasing concentrations of CmpdA for 24 h and lysed. The expression of ETS-1, p65, and β-actin was detected by Western blot analysis.

We examined whether IKK and ETS-1 could interact in Cal27CP cells. The cell lysates from Cal27CP cells were immune-precipitated with IgG control, IKKβ, or ETS-1, respectively. The immunoprecitates were detected with antibodies against IKKβ or ETS-1, followed by Western blot analysis. The results showed that IKKβ was detected in ETS-1 antibody immunoprecipates and ETS-1 was detected in IKKβ-antibody-immunoprecipated lysates (Fig. 4a). Similar results were found in SCC25CP and FaDu-CP cells (Data not shown). These results suggested an interaction between IKKβ and ETS-1 in cisplatin-resistant HNSCC. We next determined whether IKKβ regulated ETS-1 through degradation control. Cal27CP and SCC25CP cells were treated with different doses of CmpdA for 24 hours prior to treatment with either DMSO control or the protease inhibitor MG-132 for two hours. Similar to the results shown in Fig. 2, CmpdA caused dose-dependent decreases of ETS-1 expression in DMSO vehicle control-treated cells, but not in the cells treated with MG-132. As a control, the expression of p65 was not decreased by CmpdA treatment. These data indicate that IKKβ can help regulate ETS-1 degradation (Fig. 4d, e).

### Dasatinib cooperates with the IKKβ inhibitor CmpdA to inhibit ETS-1 and NF-κB, as well as to induce caspase-3 cleavage

**Fig. 5:**
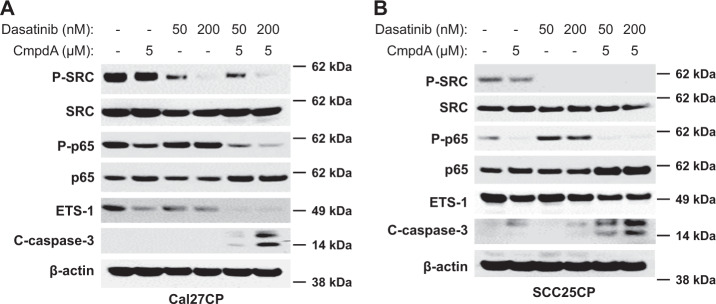
Synergistic inhibition of IKKβ/NF-κB and ETS by combination of Dasatinib with IKKβ inhibitor, CmpdA. Cal27CP (**a**) and SCC25CP (**b**) cells were treated with vehicle control, CmpdA, Dasatinib, or a combination for 24 h, lysed, and phosphorylation and total levels of SRC and p65 and expression of ETS-1, cleaved-caspase-3, and β-actin were detected by Western blot analysis.

Both NF-κB and ETS-1 are involved in cell proliferation, survival, and resistance to chemo- and targeted therapies^24–29^. We next determined whether simultaneous blockage of the SRC and IKKβ signaling pathways could lead to a significant increase in IKK/NF-κB and ETS-1 inhibition. Cal27CP cells were treated with CmpdA, Dasatinib, or a combination for 24 h. CmpdA inhibited phosphorylation of NF-κB and decreased ETS-1 expression (Fig. 5a, lane 1 versus 2). Dasatinib blocked SRC phosphorylation and decreased ETS-1 expression, while still inducing NF-κB phosphorylation (Fig. 5a, lane 1 versus lanes 2 and 4). The combination of Dasatinib and CmpdA more effectively inhibited SRC, NF-κB, and ETS-1, as well as induced significant caspase-3 cleavage (Fig. 5a, lanes 5 and 6) in comparison to either treatment alone. Similar results were found in SCC25CP cells (Fig. 5b). These results suggest that Dasatinib cooperates with the IKKβ inhibitor to inhibit ETS-1 expression and NF-κB activity, as well as induce caspase-3 cleavage.

### Dasatinib and CmpdA synergistically induce apoptosis in cisplatin-resistant HNSCC

**Fig. 6:**
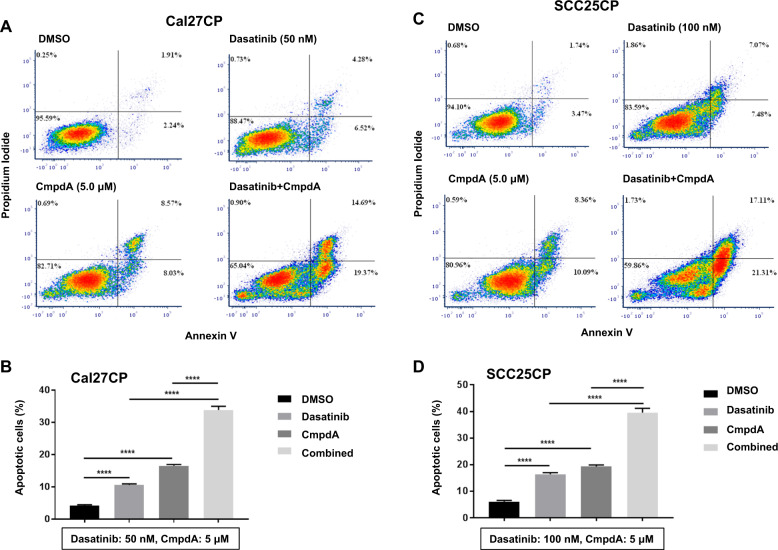
Synergistic induction of apoptosis after combination of Dasatinib with IKKβ inhibitor, CmpdA. **a**–**c** Cal27CP (**a**) and SCC25CP (**c**) were treated with vehicle control, CmpdA, Dasatinib or a combination for 48 h. Cell apoptosis was measured by Annexin V. **b**–**d** Experiments in **a** and **c** were performed in triplicate, and early and late stage apoptosis in Cal27CP (**b**) and SCC25CP (**d**) were counted and statistical analysis was performed. *P*-values <0.05 were considered to be statistically significant.

The ability of Dasatinib and CmpdA in combination to increase caspase-3 cleavage prompted us to determine the effects of Dasatinib, CmpdA, or their combination, on apoptosis. Cal27CP cell were treated with either Dasatinib, CmpdA, or a combination for 48 h, and early and late-stage apoptosis was determined by Annexin V. Treatment of cells with 100 nM Dasatinib induced apoptosis by 11%, while treatment of cells with 5 μΜ CmpdA induced apoptosis by 17%; however, the combination induced apoptosis by 35% (Fig. 6a, b). Similar experiments were performed in SCC25CP cells, and the results showed that treatment with Dasatinib or CmpdA alone induced apoptosis, whereas the combination treatment caused more (Fig. 6c, d). Our data indicate that Dasatinib and CmpdA synergistically induce apoptosis in cisplatin-resistant HNSCC.

### Inhibition of IKKβ/NF-κB to improve the efficacy of Dasatinib to suppress cisplatin-resistant HNSCC

**Fig. 7:**
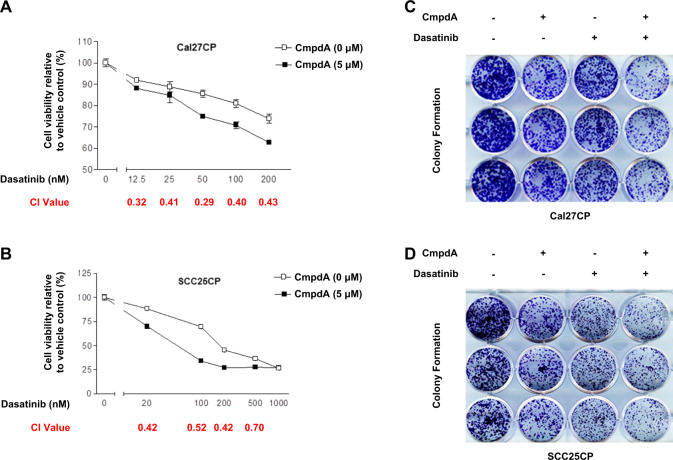
Dasatinib and CmpdA synergistically inhibit cell proliferation. **a**, **b** Dasatinib and IKKβ inhibitor, CmpdA, synergistically inhibit cell proliferation. Cal27CP (**a**) or SCC25CP (**b**) cells were treated with DMSO, Dasatinib, CmpdA, or a combination for 72 h and cell proliferation was measured by MTS assay. The experiments were performed in triplicate, and the results are representative of three independent experiments. The combination index values (CI values) were determined using CalcuSyn software. **c**, **d** Synergistic inhibition of colony formation by Dasatinib and CmpdA combination. Cal27CP (**c**) or SCC25CP (**d**) cells were treated with DMSO, Gefitinib, CmpdA, or a combination for 24 h and colony formation was observed 10 days after treatment. Each experiment was performed in triplicate.

Next, we examined whether IKKβ inhibition enhanced the ability of Dasatinib to inhibit cell proliferation. Dasatinib inhibited Cal27CP cell proliferation in a dose-dependent manner. However, addition of 5 μΜ CmpdA to Dasatinib treatment led to increased inhibition of cell proliferation (Fig. 7a). The combination index values (CI) were analyzed according to the Chou–Talalay method^30^ and the results showed that CI values from all of the combined inhibitor doses were less than 1 (Fig. 7a). Similar results from MTT assays were found in SCC25CP cells (Fig. 7b). We also performed colony formation assays in Cal27CP and SCC25CP cells. Treatment of Cal27CP cells with Dasatinib or CmpdA inhibited colony formation, but the combination of Dasatinib and CmpdA significantly increased this inhibition (Fig. 7c, d). These data indicate that CmpdA improves the efficacy of Dasatinib to inhibit cell proliferation.

## Discussion

We recently demonstrated that SRC/ETS-1 signaling was elevated in cisplatin-resistant HNSCC^20^. Depletion of ETS-1 significantly impaired cell proliferation and survival, as well as cisplatin resistance. Interestingly, inhibition of SRC by the SRC inhibitor Dasatinib only marginally diminished cell proliferation and survival. These results imply that it is important to identify the compensatory survival pathways that are up-regulated upon Dasatinib treatment.

**Fig. 8:**
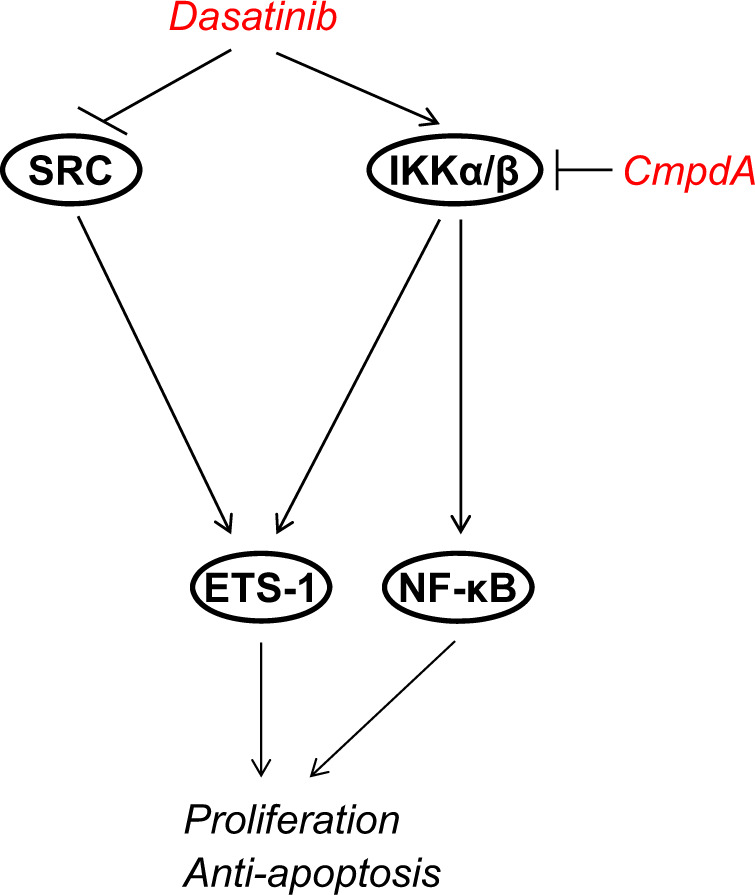
Model that illustrates pathways in response to Dasatinib and CmpdA treatment in cisplatin resistant-HNSCC cells. Dasatinib inhibited SRC activity and lowered ETS-1 expression, but induced the IKK/NF-κB pathway, while the IKKβ inhibitor CmpdA blocked Dasatinib induction of IKK/NF-κB. A combination of Dasatinib and CmpdA led to significant inhibition of ETS-1 and IKK/NF-κB as well as cell proliferation and survival.

In this study, we investigated the molecular link between SRC and the IKK/NF-κB pathway. We found that: (1) Inhibition of SRC by Dasatinib lowered ETS-1 expression but elevated the IKK/NF-κB pathway; (2) IKK interacted with ETS-1 and regulated its degradation independent of NF-κB and SRC; and (3) Combination of Dasatinib and the IKKβ inhibitor CmpdA led to significant inhibition of ETS-1 expression, cell proliferation, and cell survival (Fig. 8). Our data explored the crucial role of IKK/NF-κB in conferring resistance to SRC inhibitors in cisplatin-resistant HNSCC.

Nuclear Factor κB (NF-κB) plays important roles in the regulation of cell proliferation and survival, as well as resistance to chemo- and targeted therapies^31,32^. Many factors are involved in IKK/NF-κB activation^33–35^. Our current study showed that inhibition of SRC kinase led to up-regulation of IKK/NF-κB, but we are not currently clear on the precise mechanism(s) by which Dasatinib activates IKKβ/NF-κB pathway. Our data are consistent with the results from the study by Wolf, et al., which showed that SRC inhibition activated IKK/NF-κB to increase IL-12 synthesis on TLR-mediated activation in dendritic cells (DCs)^36^. It is important to further determine how Dasatinib can induce IKK/NF-κB and whether or not other SRC kinase family members such as Fgr, Hck, and Lyn are also involved in this process.

The transcription factor ETS-1 plays an important role in several cancers, including breast, lung, and head-and-neck^25,27,37–39^. Previous studies have shown that SRC kinase inhibits ETS-1 degradation through phosphorylation and inhibition of the tumor suppressor gene protein FRWD2 (COP1)^38^. In this study, we demonstrated that knockdown of IKKβ, but not NF-κB, decreased ETS-1 expression, but had no effect on SRC phosphorylation and expression. These data suggest that IKKβ regulates ETS-1 through mechanisms independent of SRC. It would be very interesting to define the more detailed mechanisms by which IKKβ regulates ETS-1 in cisplatin-resistant HNSCC.

The current study emphasized the functional interaction of SRC, ETS, and IKK/NF-κB to control proliferation and survival, as well as on the efficacy of combining Dasatinib with the IKKβ inhibitor, CmpdA, to inhibit cell proliferation through simultaneous inhibition of NF-κB in cisplatin-resistant HNSCC. It should be noted that Dasatinib also inhibits Bcl-Abl, which also contributes to NF-κB/IKK activation^40^. It has been reported that blockage of Bcl-Abl-induced NF-κB activation via IKKβ inhibition is effective in suppression of chronic myelogenous leukemia. It might also be important to test if a combination of the Bcr-Abl inhibitor Imatinib with CmpdA synergistically inhibits cisplatin-resistant HNSCC.

## Materials and methods

### Cell culture

HNSCC cell lines, Cal 27, FaDu, and SCC25, were obtained from ATCC. Cisplatin-resistant Cal27 cells (Cal27CP), SCC25 cells (SCC25CP) and FaDu cells (FaDu-CP) were generated from parental Cal27 and SCC25 cells through long-term treatment with cisplatin (0.5–5 μΜ) until the cells can grow normally in media with 5 μΜ cisplatin. The cell lines were authenticated by short tandem repeat analysis (STR) and tested for mycoplasma contamination in the Translational Core Facility of the University of Maryland Marlene and Stewart Greenebaum Cancer Center. All cells were cultured in Dulbecco’s modified Eagle’s medium (DMEM) supplemented with 10% fetal bovine serum (FBS), 2 mM glutamine, and 100 U/mL penicillin and streptomycin (Gibco).

### Antibodies and inhibitors

The following antibodies were purchased from Cell Signaling Technology (CST): phospho-SRC-Y416 (CST-2101), SRC (CST-2123), ETS-1 (CST-14069), phospho-p65-S536 (CST-3033), p65 (CST-6956), phospho-IKKα (S176)/β (S177) (CST-2697 and CST-2078), IKKα (CST-2682), IKKβ (CST-8943), cleaved-caspase 3 (CST-9664), and β-Actin (CST-4967). Dasatinib was from Selleck Chemicals. IKKβ inhibitor, CmdA was a gift from Dr. Albert Baldwin (University of North Carolina at Chapel Hill, Chapel Hill, NC, USA).

### Cell lysis and Western blot analysis

Cells were lysed and Western blot experiments were performed as described previously^19,41^.

### siRNA knockdown experiment

Nonspecific control siRNAs and siRNA SMARTpool IKKα, IKKβ and NF-κB (p65) were purchased from Dharmacon. Cells were transfected with non-target siRNA, and siRNA against IKKα, IKKβ, or p65 using Lipofectamine Rnaimax Transfection Reagent (Thermo Scientific) according to the manufacturer’s instructions.

### Analyzing apoptosis by Annexin V/propidium iodide staining

Cells treated with inhibitor(s) for two days were trypsinized, washed with PBS and Annexin V binding buffer, and re-suspended in 1 mL Annexin V binding buffer. 2 ×10^5^ cells were then stained with 0.5 μL of Annexin V and 0.7 μL of propidium iodide (PI) for 15 min at room temperature. Staining was then analyzed by flow cytometry on BD FACSCanto II™ Cell Analyzer (BD Biosciences). Results were analyzed by FCS Express 6. All experiments were performed in twice by triplicate and statistical analysis was performed (mean ± SD).

### Cell proliferation assays

Cell proliferation was assessed by MTS assay using the CellTiter 96 Aqueous ONE Solution kit (Promega) as described previously^41^. In brief, 5 × 10^4^ cells/mL were seeded into 96-well plates for 24 h. The next day, media were replaced with fresh media that contained the indicated concentrations of Dasatinib, CmpdA, a combination of Dasatinib and CmpdA, or the vehicle control (DMSO). After an additional 72 h incubation, MTS reagent (20 μL) was added to each well and cells with the reagent were incubated at 37°C for 2 h. Absorbance at 490 nm was measured using a microplate reader (Bio-Rad). Each experiment was performed in triplicate. In order to determine synergy of drug combination, the combination index values were determined according to the Chou–Talalay method (26) using CalcuSyn software.

### Colony formation assay

1000 cells were seeded in 12-well plates. The next day, cells were treated with vehicle control, Dasatinib, CmpdA or a combination for 48 hours, and then grown in normal media for 10 to 14 days. After gently washing once with 1× PBS, cells were fixed with methanol and stained with crystal violet.

### Statistical analysis

All data are shown as mean ± SD. Statistical analysis was performed using GraphPad Prism version 7.04 (GraphPad Software Inc.).
